# Mortality in an antiretroviral therapy programme in Jinja, south-east Uganda: a prospective cohort study

**DOI:** 10.1186/1742-6405-8-39

**Published:** 2011-10-22

**Authors:** Barbara Amuron, Jonathan Levin, Josephine Birunghi, Geoffrey Namara, Alex Coutinho, Heiner Grosskurth, Shabbar Jaffar

**Affiliations:** 1 MRC/UVRI Uganda Research Unit on AIDS, Entebbe, Uganda; 2 The AIDS Support Organisation, Old Mulago Complex, PO Box 10443, Kampala, Uganda; 3 Infectious Disease Institute, Mulago Hospital Complex, PO Box 22418, Kampala, Uganda; 4 Faculty of Epidemiology and Population Health, London School of Hygiene and Tropical Medicine, UK

**Keywords:** Antiretroviral therapy, HIV, Africa, survival, cause of death, adult, Uganda, health care delivery

## Abstract

**Background:**

There have been few reports of long-term survival of HIV-infected patients on antiretroviral therapy (ART) in Africa managed under near normal health service conditions.

**Methods:**

Participants starting ART between February 2005 and December 2006 in The AIDS Support (TASO) clinic in Jinja, Uganda, were enrolled into a cluster-randomised trial of home versus facility-based care and followed up to January 2009. The trial was integrated into normal service delivery with patients managed by TASO staff according to national guidelines. Rates of survival, virological failure, hospital admissions and CD4 count over time were similar between the two arms. Data for the present analysis were analysed using Cox regression analyses.

**Results:**

1453 subjects were enrolled with baseline median count of 108 cells/μl. Over time, 119 (8%) withdrew and 34 (2%) were lost to follow-up. 197/1453 (14%) died. Mortality rates (95% CI) per 100 person-years were 11.8 (10.1, 13.8) deaths in the first year and 2.4 (1.8, 3.2) deaths thereafter. The one, two and three year survival probabilities (95% CI) were 0.89 (0.87 - 0.91), 0.86 (0.84 - 0.88) and 0.85 (0.83 - 0.87) respectively. Low baseline CD4 count, low body weight, advanced clinical condition (WHO stages III and IV), not being on cotrimoxazole prophylaxis and male gender were associated independently with increased mortality. Tuberculosis, cryptococcal meningitis and diarrhoeal disease were estimated to be major causes of death.

**Conclusion:**

Practical and affordable interventions are needed to enable earlier initiation of ART and to reduce mortality risk among those who present late for treatment with advanced disease.

## Background

Antiretroviral therapy (ART) has been scaled-up rapidly. About 4 million people in Africa are now on ART [1]. Various studies have shown that mortality during the first 6 months or so after initiating ART is much higher than in developed countries and retention of patients in programmes is poor [2,3]. However, most longitudinal studies conducted in Africa have been either short-term or have involved small numbers of participants. A recent randomised trial conducted in Uganda and Zimbabwe comparing clinical with laboratory driven monitoring found very high and similar 5-year survival rates in the two trials arms of 87% and 90% respectively [4]. However, the participants had good access to care and were managed in research clinics by dedicated physicians. Much less is known about long-term survival on antiretroviral therapy under normal health service conditions where access to care is more difficult and the quality and level of clinical support is lower.

Understanding the temporal sequence and causes of death of patients on ART is vital to inform intervention strategies, but most deaths occur at home and cause of death is not determined. There is little understanding of the causes of death of people on ART in Africa. Here we report on the mortality rates of participants followed in an ART programme in a normal health service setting in Jinja, SE Uganda, together with the estimated causes of deaths from verbal autopsies.

## Methods

The study was based at The AIDS Support Organisation (TASO) clinic in Jinja, South East Uganda. TASO is a large non-governmental organisation (NGO) which provides care and support to people living with HIV/AIDS. Participants were enrolled in a cluster-randomised trial comparing home-based HIV care with facility care [5]. All adult patients (18 years old or more) living within the TASO catchment population and initiating on ART were invited to join and enrolled consecutively. The trial was integrated into normal health service delivery [5-7]. Trial participants were managed identically to non-trial patients by TASO staff using national guidelines.

Enrolment was done between February 2005 and December 2006 and participants were followed up till 31^st ^January 2009. The trial showed that participants in the home and facility care arms had similar rates of survival, virologic failure, CD4 count recovery, and reported adherence and the costs incurred by the health service were marginally lower in the home-care arm. Just 24/1477 (1.6%) had refused to join the trial. Details of the two arms are published elsewhere [5]. In brief, in the home-based care arm, field officers travelling on motorbikes delivered drugs to the homes of study participants and provided adherence support. Participants were assessed using a checklist and referred to the clinic if necessary. A single repeat home-visit was done for those not found at home, usually 1-2 days after the appointment date. In the facility arm, patients were asked to come monthly to clinic to pick up their drugs. On each visit they were reviewed by a nurse, received adherence counselling and were referred to a physician if necessary. Those who failed to attend clinic were usually visited at home 1-2 days after the appointment date and reminded to attend. Routine clinic visits were scheduled at 2, 6 months after starting ART and 6-monthly thereafter in the home arm and at 2, 3 and 3-monthly thereafter in the facility arm. At each of these visits, participants were reviewed by a counsellor and a physician. Participants in both arms were asked to self-refer at any time they felt unwell. They were declared as lost-to-follow-up if their whereabouts were not known on at least 2 occasions one month apart. Routine CD4 count testing was done 6-monthly for all trial participants.

Deaths were detected from TASO records or through home visits. A research physician visited the homes of the deceased, usually more than two months after the death, and collected information on the events, signs and symptoms leading up to the death. Any available medical records were also collected. Two research physicians independently reviewed the information and assigned the probable cause of death. These records were reviewed by a senior independent physician from the government referral hospital to confirm the diagnoses or to resolve discrepancies.

Participants who moved out of the area were withdrawn from the study. Their survival status was ascertained between November 2008 and January 2009 through TASO records and home visits. The trial was approved by the institutional review boards of the Uganda Virus Research Institute, London School of Hygiene and Tropical Medicine, US Center for Disease Control and Prevention and the Uganda National Council of Science and Technology.

### Statistical Analysis

Survival analysis methods were used to analyze the time to death. Subjects who did not die were regarded as being censored on the day that they were last seen or the date of withdrawal or loss to follow-up (unless it was established that a subject who withdrew or was lost to follow-up had later died, in which case they were treated as having died on the date of death recorded during tracing). In order to explore factors associated with mortality, unadjusted mortality rates together with 95% confidence limits were calculated for each level of the potential exposures and confounders. For the purpose of this descriptive analysis only, continuous covariates were converted to categorical variables.

Factors were considered as risk factors or confounders in models on substantive grounds i.e. on the basis of prior knowledge or biological plausibility [8]. The exposures considered were baseline WHO stage, baseline CD4 count, baseline weight, baseline haemoglobin, whether or not the patient was on cotrimoxazole prophylaxis at baseline, baseline log_10 _viral load and study arm. Sex and age were regarded as potential confounders of the relationship between risk factors such as CD4 count and mortality. Although the primary analysis of the cluster randomised trial showed no difference in mortality rates between the two study arms, we did investigate study arm as an exposure. In order to evaluate the effect of these explanatory variables, both unadjusted and adjusted Cox proportional hazards regression models were fitted. The models were fitted using robust standard errors to take into account the clustering of subjects in the study design. Since the number of candidate explanatory variables was small, all were included in the final model. Random effects Poisson models were then fitted as a consistency check. Age, weight, CD4 count, haemoglobin and log10 viral load were treated as continuous covariates and fractional polynomials [9] were used to test for nonlinearity (only CD4 count showed nonlinearity and a first order square root transformation fractional polynomial was found to be optimal). Continuous covariates were scaled in order to simplify interpretation of the results. Further Cox regression models were fitted treating CD4 count (again subject to a square root transformation), weight and cotrimoxazole prophylaxis as time varying covariates, to ascertain whether this changed the interpretation compared to the use of the baseline values. All analyses were carried out using Stata release 10.1 (Statacorp. 2007)."

## Results

1453 subjects were enrolled. Table 1 shows the baseline characteristics. The majority (71%) were women, the median age was 38 years and the median CD4 count was 108 cells/μl. 938 (64.6%) had a baseline viral load of 100,000 copies/ml or more and 791 (54%) were at WHO clinical stage III or IV.

**Table 1:** Baseline social and demographic characteristics of the study population

Number in study	1453
Study arm, number (%)	
Facility	594 (41)
Home	859 (59)
Women, number (%)	1031 (71)
Age in years, median (IQR)	38 (32-44)
Education level, number (%)	
No education	234 (16)
Primary	816 (56)
Secondary/tertiary	403 (28)
Clients' main occupation, number (%)	
Business/self employed/professional	615 (42)
Farmer	389 (27)
Unemployed/Housewife	434 (30)
Other	15 (1)
WHO stage, number (%)	
I	20 (1)
II	642 (44)
III	673 (46)
IV	118 (8)
CD4 cell count cells/μl at enrolment, number (%)	
< 50	444 (30)
50-99	232 (16)
100-200	654 (45)
> 200	123 (9)
Median (IQR) cells/μl	108 (35-165)
Plasma viral load copies/ml, number (%)	
< 1,000	21 (2)
1,000-9,999	40 (3)
10,000-99,999	454 (31)
100,000-999,999	832 (57)
> = 1,000,000	106
Median (IQR), copies/ml	163,224 (63,616-370,412)
On cotrimoxazole prophylaxis, number (%)	1403 (97%)
Haemoglobin g/dl, number (%) *	
≥ 11	701 (48%)
9.5 - 10.9	407 (28%)
8.0 - 9.4	242 (17%)
6.5 - 7.9	80 (6%)
< 6.5	19 (1%)
Median g/dl (IQR)	10.9 (IQR 9.5 - 12.3)
Weight, kg, number (%)	
< 50	431 (30%)
50-60	698 (48%)
> 60	317 (22%)
Mean kg (sd)	54.2 (9.2)

Haemoglobin missing for 4 subjects. Weight missing for 7 subjects.

IQR = inter-quartile range; sd = standard deviation

119 (8%) trial subjects withdrew from the trial and 34(2%) were lost to follow-up. The reasons for the withdrawals were: 53(4%) left the area for employment, 25(2%) changed to a provider closer to their home, 20(1%) could no longer afford transport money for clinic visits, 19(1%) feared disclosure of their HIV status, 1 withdrew after developing side effects to ART and 1 claimed to have been healed spiritually. The median (IQR) time to withdrawal was 350 days (147 - 609). The survival status of the participants who withdrew from the trial was as follows: three (3%) had died, 98 (82%) were on ART with various other providers, 1 (1%) was alive and not on ART and 17 (15%) were no longer in contact with TASO.

**Figure 1:**
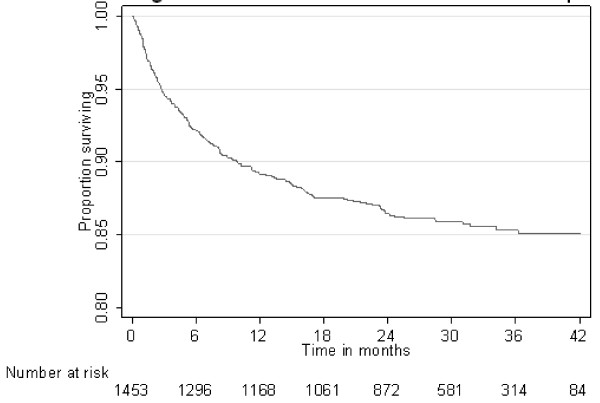
Overall survival over a 42 month duration of follow up in the study

Overall, 197/1453 (14%) trial participants died. During the first year, 154/1453 (11%) died and the mortality rate was 11.8 deaths (95% CI 10.1, 13.8) per 100 person-years. By 12 months, 1168 subjects were alive and under follow-up. Forty-three (4%) died after 12 months and the mortality rate was 2.4 deaths (95% CI 1.8, 3.2) per 100 person-years. The one, two and three year survival probabilities (95% CI) were 0.89 (0.87 - 0.91), 0.86 (0.84 - 0.88) and 0.85 (0.83 - 0.87) respectively (Figure 1). Table 2 shows the mortality rates broken down by the potential explanatory variables. Mortality was higher for males than for females, for those with baseline CD4 cell counts below 50 cells/μl, weight below 50 kg at baseline, baseline Hb below 6.5 g/dl, not on cotrimoxazole prophylaxis and increased with increasing baseline WHO stage. Mortality did not differ between the two study arms.

**Table 2:** Mortality rates by potential baseline risk factors

Factor	Level	n	Deaths	Mortality rate per 100 person-years (95% C.I.)
Sex	Male	422	69	8.0 (6.3; 10.2)
	Female	1031	128	5.7 (4.8; 6.8)

Age	≤ 30	251	41	8.7 (6.4; 11.8)
	31 to 40	670	83	5.8 (4.7; 7.2)
	> 40	532	73	6.2 (4.9; 7.7)

WHO stage	I	20	1	2.3 (0.3; 16.0)
	II	642	55	3.8 (2.9; 5.0)
	III	673	109	7.8 (6.5; 9.4)
	IV	118	32	14.9 (10.5; 21.0)

Baseline CD4 count cells/mm^3^	<50	444	110	13.0 (10.8; 15.6)
	50-99	232	20	3.9 (2.5; 6.0)
	100 - 149	306	33	4.9 (3.5; 6.9)
	150 - 199	339	21	2.8 (1.8; 4.2)
	≥ 200	132	13	4.5 (2.6; 7.7)

Baseline plasma viral load copies/ml	<1,000	21	3	6.7 (2.2; 20.7)
	1,000 - 9,999	40	2	2.6 (0.6; 10.3)
	10,000 -99,999	454	49	4.9 (3.7; 6.5)
	100,000 -999,999	832	130	7.4 (6.3; 8.8)
	>1,000,000	106	13	5.7 (3.3; 9.9)

Weight (kg) *	< 50	431	94	11.0 (9.0; 13.4)
	50 - 60	698	76	5.0 (4.0; 6.3)
	> 60	317	22	3.0 (2.0; 4.6)

Haemoglobin (g/dl)	≥ 11	701	76	5.0 (4.0; 6.3)
	9.5 - 10.9	407	64	7.4 (5.8; 9.5)
	8.0 - 9.4	242	37	7.4 (5.3; 10.2)
	6.5 - 7.9	80	12	6.7 (3.8; 11.7)
	<6.5	19	6	19.2 (8.6; 42.8)
	Missing	4	2	

On cotrimoxazole prophylaxis	Yes	1403	185	6.2 (5.3; 7.1)
	No	50	12	12.0 (6.8; 21.1)

Study Arm	Facility Based	594	80	6.5 (5.2; 8.1)
	Home Based	859	117	6.3 (5.2; 7.5)

Time following initiating ART	0 - 5 months	1453	112	16.5 (13.7; 19.8)
	6 - 11 months	1296	42	6.7 (5.0; 9.1)
	12 - 23 months	1168	32	3.0 (2.2; 4.3)
	24 - 35 months	872	8	1.3 (0.7; 2.6)
	≥ 36 months	314	3	2.4 (0.8; 7.6)

* Weight was missing for 7 people of whom 5 died.

Table 3 shows the results of fitting unadjusted and adjusted Cox regression models to find factors associated with mortality. Baseline WHO stage, CD4 count, weight, cotrimoxazole prophylaxis and gender were independent predictors of mortality. After adjusting for these factors, baseline viral load, age, Hb level and study arm were not associated with mortality. Using WHO stage II as a baseline (due to the small number of patients classified as WHO stage I), patients at WHO Stage III and IV were at increased risk. One unit increase in the transformed CD4 count led to a 39% decrease in risk of death; such a unit increase corresponds to an increase in CD4 count from 0 to 25, or from 25 to 100, or from 100 to 225; thus the effect of CD4 count is most pronounced at low CD4 counts. A 5 kg increase in baseline weight led to a 23% reduction in the risk of death. The risk of death for patients not on cotrimoxazole prophylaxis was 2.2 times the risk for patients on cotrimoxazole prophylaxis. The risk of death for females was 41% lower than the risk for males and the risk of death increased by 9% for every 10 year increase in age at baseline. The proportional hazards assumption in the Cox regression model was tested using the test of Grambsch and Therneu, and there was no evidence against the assumption (global chi-square 15.53 on 11 df, P = 0.16).

**Table 3:** Unadjusted and adjusted Cox regression models for baseline factors associated with time to death

Factor	Level	Unadjusted HR (95% c.i.)	Adjusted HR (95% c.i.)	Adjusted P-value
Sex	Male	1 (ref. level)	1 (ref. level)	0.002
	Female	0.73 (0.52; 1.02)	0.59 (0.44; 0.80)	

Study Arm	Facility	1 (ref. level)	1 (ref. level)	
	Home-based	1.003 (0.77; 1.31)	0.92 (0.70; 1.20)	0.56

Age	Per 10 year increase	1.002 (0.84; 1.20)	1.09 (0.91; 1.31)	0.30

WHO stage	I	0.58 (0.08; 4.0)	0.72 (0.12; 4.42)	0.009
	II	1 (ref. level)	1 (ref. level)	
	III	1.99 (1.57; 2.53)	1.41 (1.09; 1.83)	
	IV	3.64 (2.53; 5.23)	2.23 (1.45; 3.43)	

**√**CD4/25	Per transformed CD4 increase *	0.52 (0.44; 0.61)	0.61 (0.51; 0.73)	< 0.001

Log_10 _plasma viral load	Per 1 log increase	1.15 (1.004; 1.31)	1.04 (0.91; 1.18)	0.51

Weight	Per 5 kg increase	0.74 (0.67; 0.82)	0.77 (0.70; 0.85)	< 0.001

Haemoglobin	Per 2 g/dl increase	0.82 (0.72; 0.93)	0.93 (0.81; 1.07)	0.35

Cotrimoxazole prophylaxis	Yes No	1 (ref. level) 2.03 (1.08; 3.81)	1 (ref. level) 2.18 (1.10; 4.34)	0.02

* Because of the CD4 count transformation, per unit increases here means an increase in CD4 count from 0 to 25, or from 25 to 100, or from 100 to 225 etc;

Sensitivity analyses were done to assess the impact of including study arm in the final model and assess the effect of using random effects Poisson models, as was done in the primary analysis of the cluster randomized trial. Including study arm in the model and fitting a random effects Poisson regression had a negligible effect (e.g. the aHR for the effect of a one unit change in the transformed CD4 count changed from 0.61 in the original model to 0.60 when including study arm in the model, while the adjusted rate ratio from the random effects Poisson model was 0.59).

Overall 130/197 deaths occurred among subjects with at most one CD4 count taken at baseline. Overall, 1276 (88%) subjects had two or more CD4 counts. Of these, 1213 (95%) had increases in CD4 count and of these, 54 (4%) died. Sixty-three (5%) had a drop in CD4 count and 13 (21%) of these died. Among these 13 deaths, the median drop was 45 cells/μl (IQR 29 - 101) and the decline occurred within the first year in 8 (61.5%) of these cases. Among the 50/63 who survived, the median drop was 34 cells/μl (IQR 16 - 63) and the decline occurred within the first year in seven cases (14%). In an adjusted analyses, CD4 count fitted as a time-varying covariate only had a negligible effect (the aHR for a one unit change on the transformed scale was 0.59)

Verbal autopsies suggested that the most common causes of death identified were: CNS infections (n. 25, 13%), diarrheal disease with dehydration (n. 25, 13%), tuberculosis (n. 22, 11%) and acute febrile illness (n. 22, 11%) (Table 4). Verbal autopsies could not be done for 31 participants: relatives had moved away and could not be traced in 29 cases and there was insufficient information on which to make a diagnosis for 2 cases. There were no striking differences in the estimated causes of deaths between those which occurred within 6-months of ART initiation and which occurred later, although numbers were small.

**Table 4:** Causes of death overall and stratifed by when the death occurred after initiation of ART, number (%)

	Overall	Deaths < 6 months	Deaths ≥ 6 months
Cause of Death			

Central nervous system infections	25 (13)	19(17)	6(7)

Diarrheal disease with dehydration	25 (13)	17(15)	8(10)

Tuberculosis	22 (11)	13(12)	9(11)

Acute febrile illness	22 (11)	14(12)	8 (10)

Poor feeding/starvation	14 (7)	8(7)	6(7)

Anaemia	13 (7)	8 (7)	5 (6)

Septicemia	13 (7)	7 (6)	6 (7)

Pneumonia	7 (4)	3(3)	4(5)

Hemorrhage	5 (3)	2(2)	3(4)

Cardiovascular Disease	4 (2)	2(2)	2(2)

Kaposi's Sarcoma	4 (2)	4(4)	0(0)

Trauma/accidents	3 (2)	1(1)	2(2)

Liver failure	3 (2)	0(0)	3(4)

Suicide	2 (1)	1(1)	1(1)

Other	4 (2)	2(2)	2(2)

Unknown	31 (16)	12(11)	19(23)

Total	197	113	84

## Discussion

In this trial, which was integrated into normal health service delivery in Uganda, the mortality rate was almost 12 deaths per 100 person-years in the first year following ART initiation, five-fold higher than in subsequent years. Indeed from 12 months onwards, the mortality rate in our cohort was only slightly higher than that reported in developed countries [10]. Other studies have reported between 8 and 26% of patients dying within the first year of starting ART [2]. The mortality rate in the large DART was lower, with 5-year survival of 87% and 90% in the clinically driven monitoring versus laboratory and clinical monitoring arms respectively. However, participants in this study had better access to clinical care. The problem in Africa lies with the very high early mortality. We have previously shown an even higher mortality rate, around 30 deaths per 100 person-years, during the pre-treatment screening period [11] in this population and recent reviews show 32% loss to follow-up during this pre-treatment period despite eligibility for ART [12], a 20% loss to follow-up among patients within the first year of antiretroviral therapy [13], among whom mortality is very high [14]. Taken together, these findings suggest that the major problem is early mortality and that mortality after 12 months in well functioning programmes will be low. We believe our findings are robust. Very few individuals refused to join the study, the loss-to-follow-up was low and the mortality rate among subjects who withdrew was similar to those who were retained in care.

Patients in Africa generally present late for HIV treatment with CD4 count typically well below 200 cells per microlitre [15], as was the case in our study. Much of the mortality in our study was attributed to tuberculosis, cryptococcal meningitis and diarrhoea and clearly some of this could have been prevented had patients initiated ART earlier. However earlier ART initiation will not be a reality for many because of the costs and difficulties of accessing care, particularly for chronic conditions such as HIV which require regular contact with health services. We have previously shown that a single visit to the clinic in our area costs 13% of a man's and 20% of woman's monthly salary and that the cost of transport is a major factor inhibiting patients starting ART [5]. There is therefore an urgent need to identify practical interventions which will reduce early mortality among the many patients who will continue to present with advanced HIV associated disease for the foreseeable future.

Our study also identified several factors associated independently with mortality. As expected low baseline CD4 count and WHO clinical stage III or IV were associated independently with increased mortality; but there was no association with plasma viral load. Most patients who died had only one CD4 count measured at baseline. Among those in whom CD4 count was measured more than once, mortality risk was more than 5-fold higher among patients who had falls in their CD4 count than those in whom CD4 count increased. However, fitting CD4 count as a time-varying covariate did not significantly improve the fit of the model compared to fitting CD4 count at baseline. Although our numbers were small, this accords with the findings of the DART trial [4] which showed limited additional value of CD4 count monitoring over clinical monitoring. More research is needed to assess the costs and benefits of long-term CD4 count monitoring in settings where access to care is limited.

Men presented at a similar WHO stage and CD4 count as women but had significantly higher plasma viral loads. After adjusting for these and the other factors including whether on cotrimoxazole prophylaxis and weight, men had a much higher mortality than women. Why this is so is puzzling. We know that in most settings, fewer men access antiretroviral services [13,16]. One explanation for their higher mortality could be that men maintain less contact with health services and have poorer adherence to treatment. This needs further investigation in other cohorts. If true, it points to a poor prognosis for men: fewer start antiretrovirals and those who do may have poorer outcomes.

In conclusion, our study shows that under conditions of normal health services the high early mortality of HIV infected patients starting antiretroviral therapy is associated with low baseline CD4 count, body weight, advanced clinical conditions (WHO stages III and IV), not being on cotrimoxazole prophylaxis and male gender. Practical and affordable interventions are needed to enable earlier initiation of ART and to reduce mortality risk among those who present late for treatment with advanced disease.

## Competing interests

The authors declare that they have no competing interests.

## Authors' contributions

BA and SJ wrote the first draft of the paper and all authors contributed comments and suggestions to various draft versions. JL conducted the statistical analyses and GN managed the data. JB was responsible for patient management and JB, BA, GN were responsible for study implementation. AC, HG and SJ generated the research ideas, secured funding and co-ordinated the research programme All authors have seen and approved the final version.
